# Genetic Epidemiology and Resistance Investigations of Clinical Yeasts in Alexandria, Egypt

**DOI:** 10.3390/pathogens14050486

**Published:** 2025-05-15

**Authors:** Bram Spruijtenburg, Carolina Melchior do Prado, Mats van Kempen, Sherine M. Shawky, Jacques F. Meis, Vânia Aparecida Vicente, Flavio Queiroz-Telles, Theun de Groot, Mohammed A. El-Kholy, Eelco F. J. Meijer

**Affiliations:** 1 Radboudumc-CWZ Center of Expertise for Mycology, 6532 SZ Nijmegen, The Netherlands; 2 Department of Medical Microbiology and Immunology, Canisius-Wilhelmina Hospital (CWZ)/Dicoon, 6532 SZ Nijmegen, The Netherlands; 3 Postgraduate Program in Microbiology, Parasitology and Pathology, Biological Sciences, Department of Basic Pathology, Federal University of Paraná, Curitiba 82590-300, Brazil; 4 Department of Microbiology, Medical Research Institute, Alexandria University, Alexandria 5422021, Egypt; 5 Institute of Translational Research, Cologne Excellence Cluster on Cellular Stress Responses in Aging-Associated Diseases (CECAD) and Excellence Center for Medical Mycology, University of Cologne, 50931 Cologne, Germany; 6 Laboratory of Microbiology and Molecular Biology, Department of Basic Pathology, Federal University of Paraná, Curitiba 82590-300, Brazil; 7 Department of Public Health, Hospital de Clínicas, Federal University of Paraná, Curitiba 80060-900, Brazil; 8 Department of Microbiology and Biotechnology, Clinical and Biological Sciences Division, College of Pharmacy, Arab Academy of Science, Technology and Maritime Transport (AASTMT), Alexandria 1029, Egypt

**Keywords:** *Candida*, candidiasis, candidemia, epidemiology, antifungal resistance, genotyping, short tandem repeats

## Abstract

Yeast bloodstream infections lead to high mortality and morbidity and are mostly observed in immunocompromised patients. In Africa, only a few studies have characterized clinical yeasts. To increase insight into yeast resistance and transmission in Africa, we identified various yeasts from Alexandria, Egypt and performed antifungal susceptibility testing (AFST) and genotyping. A total of 1307 single isolates from unique patients, recovered from different anatomical sites including the bloodstream, retrieved from a reference laboratory in Alexandria, Egypt were studied. All isolates were identified with MALDI-TOF MS, while some were initially identified with a Vitek 2 Compact system. Short tandem repeat (STR) genotyping was performed for the most common species, and AFST was performed with microbroth dilution. Among bloodstream isolates (*n* = 71), *C. albicans* was the most common etiological agent, followed by *C. tropicalis* and *C. parapsilosis*. Comparison of yeast identification methods demonstrated that 22% of isolates were incorrectly identified with the Vitek 2 Compact system compared to MALDI-TOF MS. Multiple rare yeasts showed reduced antifungal susceptibility. STR genotyping demonstrated potential events of nosocomial transmission with *N. glabratus* and *C. parapsilosis*. Moreover, an azole-resistant *C. tropicalis* clade identified earlier in Alexandria was still present. To conclude, clinical yeasts in Alexandria, Egypt, are overall susceptible common species.

## 1. Introduction

Most yeast species are human commensals of the skin, oral cavity, and gastrointestinal tract, with common *Candida* yeasts detected in up to 60% of healthy humans [1]. Despite yeasts being part of a healthy microbiome, colonization is a prerequisite for infection, which occurs in patients with severely weakened immunity or through the usage of antibiotics, among other risk factors [2]. Yeast infections present with different clinical manifestations, with vulvovaginal candidiasis (VVC) as one of the most common, affecting over 75% of women at least once during their life [3]. Single episodes of VVC are easily cured, but recurrent VVC needs to be treated with systemic azole therapy [3]. Candiduria is defined as the presence of yeasts in urine, is often observed in intensive care unit (ICU) patients, and mostly reflects colonization [4]. Although yeasts in the urinary tract are often cleared without antifungal treatment, they can be a focus for resistance development [4]. Severe disease occurs when yeasts enter the bloodstream, defined as candidemia, with mortality rates around 50% [5]. Early detection followed by appropriate antifungal therapy is warranted for a good clinical outcome; however, this remains a major challenge in low- and middle-income countries [6]. Globally, *Candida albicans*, *Nakaseomyces glabratus* (also known as *Candida glabrata*), *Candida parapsilosis*, *Candida tropicalis*, and *Pichia kudriavzevii* (previously known as *Candida krusei*) are the most common species causing bloodstream infections, but this varies based on country and patient population [5]. For example, in Latin America candidemia is characterized by a high prevalence of *C. tropicalis* and *C. parapsilosis*, while in Western Europe *C. albicans* and *N. glabratus* are more prevalent [7,8]. Additionally, antifungal resistance rates are variable, as *C. albicans* is overall susceptible, while other species like *C. parapsilosis* are often resistant to one or multiple antifungals and capable of causing nosocomial outbreaks [8,9]. Therefore, species identification and antifungal susceptibility testing (AFST) are essential to guide patient management with adequate antifungal stewardship.

Africa is highly underrepresented with regards to medical mycology, with a limited number of laboratories having access to accurate species identification, AFST, and appropriate antifungals [10]. South Africa is one of the few countries in which comprehensive epidemiological candidemia studies have been conducted. Alarmingly high rates of resistant *C. parapsilosis* and *Candida auris* were found [11]. In contrast, candidemia studies from North Africa found limited antifungal resistance, while species distribution differed between countries, with *C. tropicalis* showing the highest prevalence in Tunisia, and *C. albicans* in Morocco [12,13]. To date, most studies from Egypt include a limited number of cases and often lack accurate species identification [14,15], highlighting the need for additional studies. To this end we identified, determined the antifungal susceptibility of, and genotyped a large collection of yeasts from patients in Alexandria, Egypt.

## 2. Materials and Methods

### 2.1. Isolates and Identification

A total of 1307 yeast isolates from unique patients were collected during two periods in Alexandria, Egypt. During the first period, from 1 June 2022 to 18 January 2023, all yeasts (*n* = 345) that were included were identified with a Vitek 2 Compact system (BioMérieux, Marcy-l’Etoile, France), mostly based on clinical relevance. These isolates were used for data analysis and, with exception of *C. albicans* (*n* = 183), also identified by matrix-assisted laser desorption ionization-time-of-flight (MALDI-TOF) mass spectrometry (MS), as previously described [16]. From 19 January 2023 to 31 October 2023, all cultured yeasts (*n* = 962) were included in the data analysis and identified via MALDI-TOF MS, with the exception of *C. albicans* (*n* = 154), which were identified using a Vitek 2 Compact system. All cultured 162 non-*albicans* yeasts were also identified using a Vitek 2 Compact system at an earlier stage. Isolates were stored at −70 °C according to standard procedures and grown on Sabouraud agar plates (SDA) (Oxoid, Basingstoke, UK) at 35 °C prior to identification, AFST, and DNA extraction. This study was approved by the ethical committee of the Arab Academy for Science, Technology and Maritime Transport (AASTMT), under registration code AASTMT-ERC-2022-049.

### 2.2. Short Tandem Repeat (STR) Genotyping

Multiplex PCR STR genotyping was performed for *C. tropicalis*, *C. parapsilosis*, *N. glabratus*, *C. auris*, and *P. kudriavzevii*, as previously described [17,18,19,20]. *C. auris* STR profiles were compared to isolates from other clades [21]. In short, for STR genotyping, DNA was extracted by suspending cells in 50 µL of MightyPrep Reagent for DNA (Takara Bio Inc., Shiga, Japan), followed by incubation at 95 °C for 10 min. The cells were spun down at high speed for 2 min, after which the supernatant was taken for amplification. Multiplex PCR, amplifying three microsatellite markers each, was conducted using the Terra™ PCR Direct Polymerase Mix (Takara Bio Inc.), consisting of 1× Terra PCR Direct Buffer, forward and reverse primers (0.1–1 µM), 1.25 U Terra PCR Direct Polymerase Mix, 1.5 µL of isolated DNA, and 12.5 µL of sterile water in a final volume of 25 µL. Amplicons were diluted 1000 times, and 0.12 µL Orange-600 DNA Size Standard (Nimegen, Nijmegen, The Netherlands) was added. These diluted products were run on a 3500 XL genetic analyzer (Applied Biosystems, Foster City, CA, USA), and copy numbers were determined using GeneMapper 5 software (Applied Biosystems). Relatedness between isolates was analyzed with BioNumerics software v7.6.1 (Applied Maths NV, Sint-Matems-Latem, Belgium), as described previously [18].

### 2.3. Resistance Investigation

Susceptibility testing against eight common antifungals was performed by broth microdilution according to the Clinical and Laboratory Standards Institute (CLSI) M27-S4 guideline [22]. In short, cultures were diluted to a final concentration of 1 × 10^3^ CFU/mL in RPMI 1640 medium. Minimum inhibitory concentrations (MICs) were read visually after 24 h of incubation at 35 °C as the lowest antifungal concentration with a 50% growth reduction as compared to the growth control, except for amphotericin B, for which 100% growth reduction was used. For fluconazole-resistant *C. tropicalis* isolates, the full ERG11 gene was amplified, as described previously [23]. Amplicons were purified with Ampliclean and D-Pure protocols (Nimagen, Nijmegen, The Netherlands), followed by Sanger sequencing on a 3500 XL genetic analyzer (Applied Biosystems).

## 3. Results

### 3.1. Species Distribution

**Figure 1:**
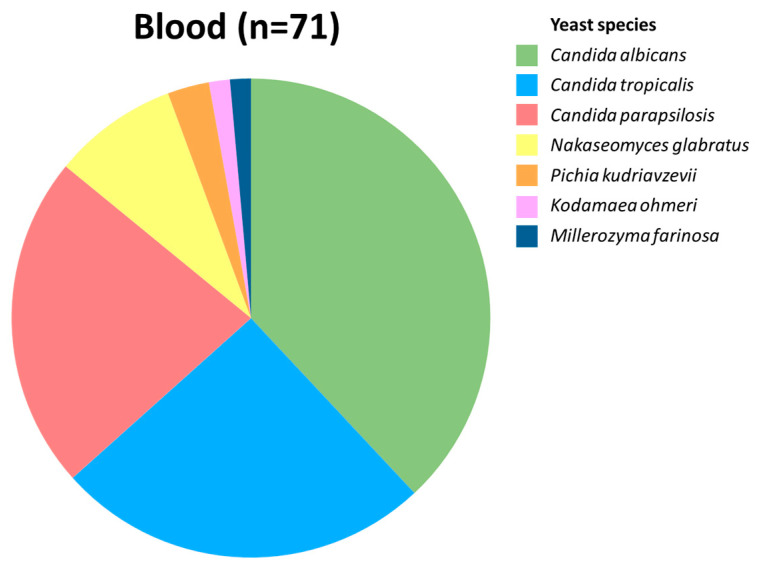
Species distribution of candidemia cases (*n* = 71).

**Figure 2:**
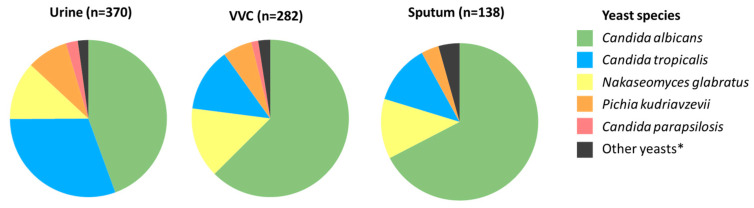
Distribution of yeast species from non-sterile sources. * Other yeasts include for VVC two *Pichia cactophila*, one *Candida metapsilosis*, and one *Candida orthopsilosis* isolate, for urine four *Kluveromyces marxianus*, three *Candida dubliniensis*, one *Clavispora lusitaniae*, one *P. cactophila*, one *Diutina rugose*, and one *Saccharomyces cerevisiae* isolate, and for sputum two *C. orthopsilosis*, two *K. marxianus*, and one *P. cactophila* isolate. VVC, vulvovaginal candidiasis.

During a 17-month period (1 June 2022 to 31 October 2023), 71 yeasts were isolated from blood and collected by the Microbiology unit of Mabaret Al Asafra Laboratory in Alexandria, Egypt, which serves as the reference laboratory for many hospitals in the city. These isolates were subsequently identified with MALDI-TOF MS, with exception of *C. albicans* isolates, which were only identified using a Vitek Compact 2 system, as it generally correctly identifies this species [24]. Of the 71 isolates, *C. albicans* was the dominant etiological agent, followed by *C. tropicalis*, *C. parapsilosis*, *N. glabratus*, and *P. kudriavzevii* (Figure 1). Also, there were two patients with bloodstream infections involving the rare yeasts *Millerozyma farinosa* and *Kodamaea ohmeri*. In addition to the blood isolates, we aimed to comprehend the epidemiological situation by also collecting non-blood yeast isolates. From 1 June 2022 to 18 January 2023, only yeast isolates identified locally via a Vitek Compact 2 system were included, while, to obtain a complete overview, all yeast isolates from 19 January 2023 to 31 October 2023 were included. In total, 1236 non-blood yeast isolates were collected, of which most originated from vaginal swabs from women with VVC symptoms (*n* = 282, 23%), urinary tract samples (*n* = 370, 30%), and sputum (*n* = 138, 11%). Identification of these isolates was performed as described earlier. In vaginal swabs, *C. albicans* accounted for the majority (*n* = 177, 63%) of all isolates, followed by *N. glabratus*, *C. tropicalis*, and *P. kudriavzevii*, while only four *C. parapsilosis* isolates were found (Figure 2). A similar distribution was found in sputum and urine, although in the latter *C. tropicalis* together with *C. albicans* was found most often. The remaining sample sources (*n* = 15) were too small (<20 isolates) to interpret species distribution. A complete overview of all yeast species from the entire period (*n* = 1307) is presented in Table S1. Besides the two cases of candidemia caused by *M. farinosa* and *K. ohmeri*, identified rare yeasts included three *Clavispora lusitaniae*, two *Candida auris*, two *Pichia cactophila*, one *Magnusiomyces capitatus*, one *Saccharomyces cerevisiae*, and one *Diutina rugosa* isolate, all from either urine or sputum cultures, likely reflecting colonization. Also, one *K. ohmeri*, two *P. cactophila*, and one *Candida metapsilosis* isolate were cultured from vaginal swabs.

### 3.2. Yeast Identification with Vitek 2 Compact System

In order to determine the accuracy of Vitek 2 Compact system yeast identification, all non-*albicans* Vitek 2 Compact system identifications (*n* = 325) obtained during the complete 17-month period and performed mostly on clinically relevant cultures were compared to MALDI-TOF MS identification. From the 325 non-*albicans* identifications performed with the Vitek 2 Compact system, 71 (22%) isolates were incorrect, using MALDI-TOF MS as the gold standard. Misidentification was frequently observed for yeasts identified by the Vitek 2 Compact system as *C. parapsilosis* and with most rare species (Table 1).

### 3.3. STR Genotyping of Non-Albicans Yeasts

**Figure 3:**
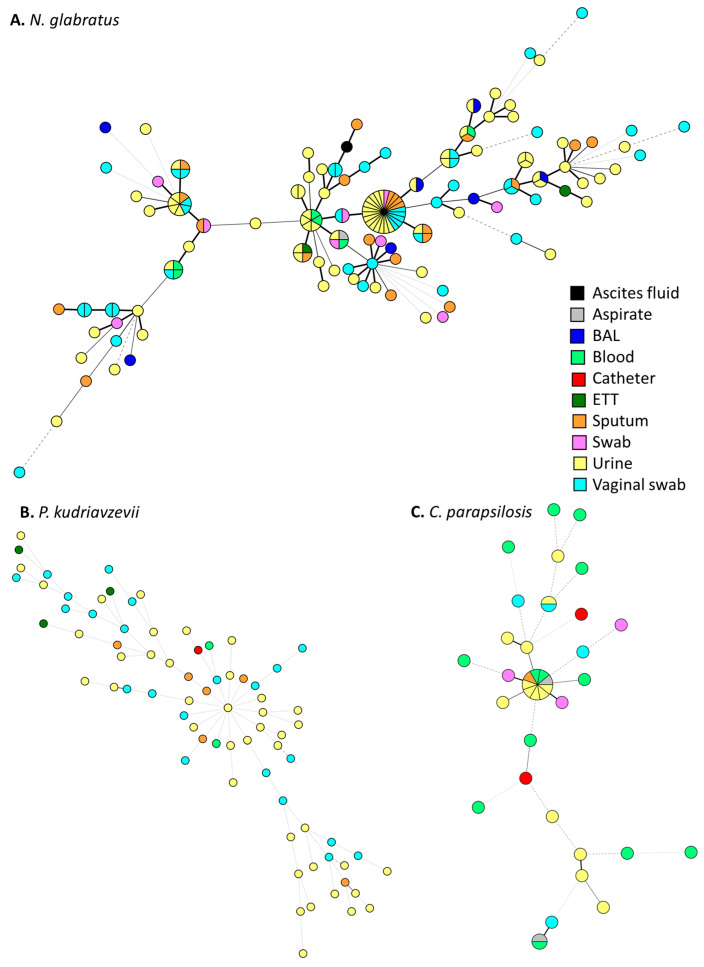
Minimum-spanning tree of 166 *Nakaseomyces glabratus* isolates (**A**), 75 *Pichia kudriavzevii* isolates (**B**) and 39 *Candida parapsilosis* (**C**) isolates. Isolates are colored according to the sample source. Branch lengths of the tree indicate genetic relatedness, with thick solid lines (variation in one marker), thin solid lines (variation in two markers), thin dashed lines (variation in three markers), and thin dotted lines (variation in four or more markers). BAL, bronchoalveolar lavage; ETT, endotracheal tube.

The phylogenetic relatedness between all available isolates from several yeast species, i.e., *N. glabratus* (*n* = 166), *P. kudriavzevii* (*n* = 75), *C. parapsilosis* (*n* = 39), *C. tropicalis* (*n* = 340), and *C. auris* (*n* = 2), were assessed with species-specific STR assays, confirming the results obtained with MALDI-TOF MS. *N. glabratus* genotyping yielded 145 unique genotypes, consisting of 1 to 24 isolates (Figure 3A). Notably, 13 large clusters (greater than or equal to three isolates) were found, and two of these contained multiple bloodstream isolates. The largest cluster comprised 24 isolates from non-sterile sites like urine, vaginal swabs, and sputum. STR genotyping of the *P. kudriavzevii* isolates yielded only unique genotypes, with differences in at least two alleles, indicating the absence of nosocomial transmission and the presence of high genetic diversity (Figure 3B). STR genotyping of *C. parapsilosis* isolates revealed three clusters consisting of two or more isolates, indicating potential events of nosocomial transmission (Figure 3C). Furthermore, there were three isolates that were closely related to two of these clusters, differing by one STR marker only. Both small clusters of two isolates comprised an isolate from blood and one from a non-sterile source. One of these small clusters consisted of two isolates from the same hospital, while isolates from the other small cluster originated from two different hospitals (Figure S1). The cluster of nine isolates comprised samples from four different hospitals.

**Figure 4:**
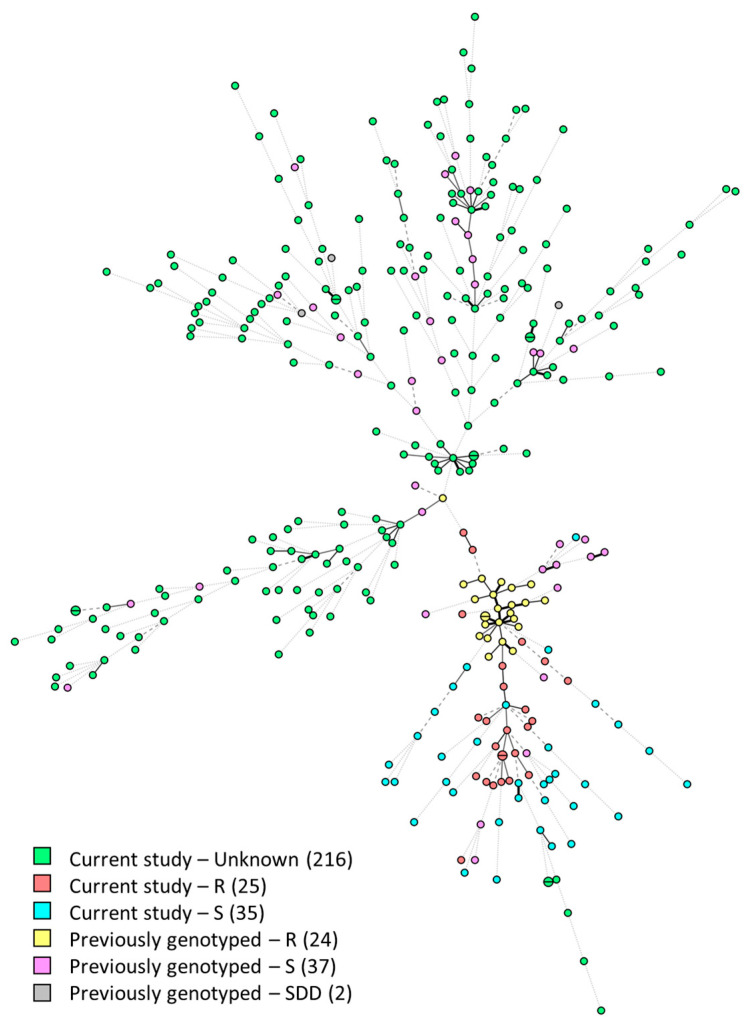
Minimum-spanning tree of 340 *Candida tropicalis* isolates. Isolates are colored according to the study in which they were typed and their fluconazole susceptibility. Branch lengths of the tree indicate genetic relatedness, with thick solid lines (variation in one allele), thin solid lines (variation in two alleles), thin dashed lines (variation in three alleles), and thin dotted lines (variation in four or more alleles). R, resistant; S, susceptible; SDD, susceptible–dose-dependent.

A total of 276 *C. tropicalis* isolates from the current study were investigated with STR genotyping and compared to 64 previously genotyped isolates from Alexandria originating from 2014 to 2015 [25]. Out of the 276 isolates, a total of 270 genotypes were found, each comprising one or two isolates. None of these genotypes was identical to one of the previously reported genotypes (Figure 4). As the collection from 2014 to 2015 constituted a fluconazole-resistant clade caused by a G464S mutation in *ERG11* [25], we performed AFST on isolates that were relatively closely related to this resistant clade, with differences in three STR markers at most. From the 60 isolates that were analyzed, 25 isolates were fluconazole-resistant, and subsequent *ERG11* sequencing also demonstrated the G464S mutation in these isolates. Finally, in the current culture collection, two *C. auris* isolates were present, collected from sputum and urine. STR genotyping of these isolates showed different genotypes, although both isolates allocated to clade I (Figure S2).

### 3.4. Resistance Investigation of Blood Culture Isolates and Rare Species

In vitro AFST with CLSI microbroth dilution was performed on blood isolates and rare species. All former isolates were found to be susceptible when breakpoints were available, except for one *C. parapsilosis* isolate (#107) that was susceptible–dose-dependent (SDD) for fluconazole and susceptible for the other tested antifungals (Table S2). Although breakpoints are often not available for rare species, elevated MICs were found for both *C. auris* isolates for fluconazole (MICs of 16 and 32, respectively). Other notable observations included elevated MICs of a *D. rugosa* isolate and a *K. ohmeri* isolate with regard to fluconazole and echinocandins, *C. orthopsilosis* to echinocandins, *P. cactophila* with regard to fluconazole, and *M. capitatus* for echinocandins (Table S3).

## 4. Discussion

Bloodstream infections by yeasts are globally leading to a million deaths every year, while in the last years an increase in antifungal resistance and shift in species distribution has been observed [26]. Unfortunately, African studies regarding yeast epidemiology are rare, while these are highly needed to inform local healthcare practices and allow antifungal stewardship [10,27]. In this study we identified a large collection of yeast isolates from sterile and non-sterile sites from patients in Alexandria, Egypt. In addition, the genetic relatedness of common species was assessed with STR genotyping, and AFST was performed on bloodstream isolates and rare species.

### 4.1. Identification of Yeasts Using a Vitek 2 Compact System and Antifungal Resistance

Accurate species identification with MALDI-TOF MS is often not available in resource-limited countries like Egypt. Instead, alternative platforms such as the Vitek 2 Compact system are frequently implemented [28]. Therefore, all non-*albicans* yeasts (*n* = 325) were also analyzed by MALDI-TOF MS, as *C. albicans* is mostly identified correctly by the Vitek 2 Compact system [24]. Using MALDI-TOF MS as the gold standard, we found that most common yeasts were correctly identified, except for *C. parapsilosis*, with 41% misidentification. Rare yeasts (*n* = 32) were commonly misidentified (81%), as also reported in other studies [24]. In addition to identification, isolates collected from blood and rare species were tested for antifungal resistance, showing only SDD *C. parapsilosis* for fluconazole and elevated MICs for some rare species. Especially for *C. parapsilosis*, the absence of azole resistance is remarkable, given that other countries report resistance rates exceeding 25% [29]. Fluconazole MICs were high for *C. auris*, *M. farinosa*, *D. rugosa*, *K. ohmeri*, and *P. cactophila*, which is more often reported [30]. Because fluconazole is often used to treat candidemia in low- and middle-income countries, the misidentification of rare yeasts as common fluconazole-susceptible yeasts, as was observed for the *K. ohmeri* candidemia, may increase the chance of treatment failure [31]. Lastly, *D. rugosa* and *M. capitatus* had elevated echinocandin MICs, which is in line with previous reports, as both species display intrinsically elevated MICs [31].

### 4.2. Species Distribution

In regard to candidemia episodes, *C. albicans* was the most frequent causative agent, followed by common species like *C. tropicalis*, *C. parapsilosis*, *N. glabratus*, and *P. kudriavzevii*. These five pathogens are often reported in other studies, although their proportion differs between and within countries [5,15,32]. A similar distribution was found by a recent Egyptian study from Cairo involving 90 patients with candidemia, which also reported *C. albicans* as the most common agent, closely followed by *C. tropicalis* and *C. parapsilosis* [15]. In contrast to our study, *N. glabratus* was nearly absent, while three episodes of *C. auris* candidemia were reported. The differences for *N. glabratus* might be due to differences in patient populations, as infections by this species mainly occur in elderly patients [33]. Unfortunately, information regarding the age of the patients was not available. The differences in *C. auris* candidemia episodes is likely due to different travel histories of patients, as infections are often imported [2]. Other North African countries also reported a large proportion of *C. tropicalis* and *C. albicans* in bloodstream infections, with *C. auris* infrequently reported, which could be due to limited diagnostic resources [12,13]. Two notable cases of infection by rare yeasts were observed, caused by *K. ohmeri* and *M. farinosa*, respectively. While *K. ohmeri* bloodstream infections are occasionally reported in Asia, America, and Europe, Tunisia is the only African country to date that has reported infections with this yeast [34]. Candidemia with *M. farinosa* is exceptionally rare, with only four bloodstream infections reported earlier, to the best of our knowledge [35]. Given the retrospective nature of this study, clinical details could unfortunately not be retrieved; however, other reported *M. farinosa* cases are often catheter-related, and echinocandins were used to successfully treat the infection.

For VVC isolates, *C. albicans* was the dominant species, comprising about two-thirds of the samples, followed by other common yeasts and a few rare species. Although a shift has been observed in bloodstream infections from *C. albicans* to non-*albicans* yeasts, *C. albicans* remains the dominant agent of VVC, as found here and in other studies [3]. Although clinically relevant species and also rare yeasts like *P. cactophila*, *D. rugosa*, and *K. marxianus* were found in urine and sputum, these likely reflect colonization, although these species can cause breakthrough infections under specific circumstances [30].

### 4.3. Genetic Relatedness as Assessed by STR Genotyping

Using STR genotyping, the genetic relationship between isolates of several species was investigated. Multiple clusters were found among *N. glabratus* isolates, including from the bloodstream. These clusters might represent dominant environmental genotypes acquired by the community or reflect the nosocomial transmission that is occasionally reported for *N. glabratus* [36]. Whole-genome sequencing (WGS) is required to reveal the underlying cause of these identical genotypes. Similarly, multiple clusters were also identified among *C. parapsilosis* isolates, suggesting potential clonal expansion. This yeast is known to frequently cause outbreaks in healthcare settings, especially when infection prevention measures are suboptimal [37,38]. Nonetheless, WGS should also be performed here to determine whether nosocomial transmission actually occurred [39]. For *P. kudriavzevii*, no clusters were found, indicating high genetic diversity and an absence of nosocomial transmission, which is in line with previous investigations [40]. Finally, all *C. tropicalis* isolates collected in the current study were genotyped. Only small clusters of two isolates were found, indicating that nosocomial transmission is rare, as was recently found in large Italian and Latin American studies [41,42]. For example, the largest clusters did not exceed 10 isolates, and usually below 5% of all isolates displayed identical genotypes. In addition, the current genotypes were compared to isolates collected from 2014 to 2015 in the same city, which comprised a fluconazole-resistant clade with the *ERG11^G464S^* mutation [25]. Current isolates that related to some extent to this clade were subsequently analyzed by AFST, which showed that 25 of these isolates were fluconazole-resistant and also harbored the same *ERG11^G464S^* mutation as found in 2014–2015. Although fluconazole resistance severely limits treatments option in resource-limited countries like Egypt, which frequently treat empirically with fluconazole, isolates of the resistant clade have not been recovered from blood in this or the earlier study. This might be due to reduced virulence, as this mutation in the *ERG11* gene of *C. albicans* was previously shown to be associated with a fitness cost [43]. Given that the clade has at least been present in Alexandria for nearly a decade, isolates seem to thrive in the environment, thereby retaining its resistance. Finally, both *C. auris* isolates were genotyped and were found to have two distinct genotypes that indicate independent introductions in the city. Both isolates allocated to clade I, which has been reported previously in other African countries, just like isolates from clades II, III, and IV [27,44]. Although *C. auris* is highly capable of clonal transmission in hospitals, the absence of clusters suggests the yeast has an overall low prevalence in this city and other Egyptian cities [15,45]. Underdiagnoses and limited availability of advanced diagnostics methods may, however, contribute to missed detections. Continued surveillance is warranted to prevent future outbreaks.

A limitation of this study is that isolates identified with the Vitek 2 Compact system as *C. albicans* were subsequently not confirmed by MALDI-TOF MS. Although the Vitek 2 Compact system identifies common yeasts correctly [24], some of these may be misidentified, so the exact proportion of *C. albicans* per anatomical source is probably slightly lower. Secondly, given the retrospective nature of this study, clinical data from the patients could not be retrieved, which made comparisons based on hospitals, wards, age groups, and sexes impossible to conduct. Last, given that the COVID-19 pandemic increased the incidence of candidemia episodes [46], the current number of cases could be higher than usual, although studies for a longer period are needed to asses this.

## 5. Conclusions

To summarize, we investigated a large collection of clinical yeast isolates from sterile and non-sterile sites from patients in Alexandria, Egypt. *C. albicans* was found to be the most common agent of bloodstream infections, closely followed by *C. tropicalis* and *C. parapsilosis*. The rare candidemia pathogens included *M. farinosa* and *K. ohmeri*. Of concern, these and other rare species were often misidentified with the Vitek 2 Compact system. STR genotyping suggested several events of potential nosocomial transmission of *N. glabratus* and *C. parapsilosis*. Antifungal resistance appears to be rare, although continued surveillance is needed.

## Figures and Tables

**Table 1:** Non-*albicans* species identification with the Vitek 2 Compact system compared to MALDI-TOF MS as the gold standard.

Species According to Vitek	*n* Total	*n* Incorrect (%)	Species Misidentified (*n*)
*Candida tropicalis*	167	18 (11)	*N. glabratus* (8), *C. parapsilosis* (3), *C. albicans* (2), *P. kudriavzevii* (2), *C. orthopsilosis* (1), *K. ohmeri* (1), *M. capitatus* (1)
*Nakaseomyces glabratus*	64	5 (8)	*C. albicans* (3), *C. tropicalis* (2)
*Candida parapsilosis*	37	15 (41)	*C. tropicalis* (8), *C. albicans* (3), *C. orthopsilosis* (2), *C. lusitaniae* (1)
*Pichia kudriavzevii*	20	3 (15)	*C. tropicalis* (1), *C. parapsilosis* (1), *N. glabratus* (1)
*Clavispora lusitaniae*	5	3 (60)	*C. tropicalis* (2), *C. parapsilosis* (1)
*Candida dubliniensis*	4	4 (100)	*P. kudriavzevii* (2), *N. glabratus* (2)
*Meyerozyma guilliermondii*	4	4 (100)	*C. tropicalis* (2), *C. albicans* (1), *C. parapsilosis* (1)
*Kluyveromyces marxianus*	4	2 (50)	*C. tropicalis* (2)
*Kluyveromyces lactis*	4	4 (100)	*K. marxianus* (4)
*Stephanoascus ciferii*	3	3 (100)	*C. albicans* (2), *C. tropicalis* (1)
*Candida auris*	2	0 (0)	NA
*Pichia norvegensis*	2	2 (100)	*P. kudriavzevii* (1), *P. cactophila* (1)
*Debaryomyces hansenii*	1	1 (100)	*C. albicans* (1)
*Millerozyma farinosa*	1	1 (100)	*N. glabratus* (1)
*Cryptococcus laurentii*	1	1 (100)	*C. tropicalis* (1)
*Zygosaccharomyces* sp.	1	1 (100)	*N. glabratus* (1)

NA, non-applicable.

## Data Availability

Data presented in the current study are available within the article and Supplementary Materials.

## Supplementary Materials

The following supporting information can be downloaded at: https://www.mdpi.com/article/10.3390/pathogens14050486/s1, Figure S1: Minimum-spanning tree of 39 *Candida parapsilosis* isolates. Isolates are colored according to the hospital. Branch lengths of the tree indicate genetic relatedness, with thick solid lines (variation in one marker), thin solid lines (variation in two markers), thin dashed lines (variation in three markers), and thin dotted lines (variation in four or more markers). Figure S2: Minimum-spanning tree of two *Candida auris* isolates with control isolates from all six known clades. Isolates are colored according to the clade or country of origin. Branch lengths of the tree indicate genetic relatedness with thick solid lines (variation in one marker), thin solid lines (variation in two alleles), thin dashed lines (variation in three markers), and thin dotted lines (variation in four or more alleles). Table S1: Isolate overview of 1307 clinical yeast isolates studied. Table S2: Minimum inhibitory concentrations (MICs) according to CLSI M27-S4 guideline of all individual 47 bloodstream isolates. MICs are displayed in µg/mL. Table S3: Minimum inhibitory concentrations (MICs) according to the CLSI M27-S4 guideline of 37 rare yeast isolates. MICs are displayed in µg/mL.

## Abbreviations

The following abbreviations are used in this manuscript:

AFST	Antifungal susceptibility testing
CLSI	Clinical and Laboratory Standards Institute
ICU	Intensive care unit
MALDI-TOF MS	Matrix-assisted laser desorption ionization-time-of-flight mass spectrometry
STR	Short tandem repeat
VVC	Vulvovaginal candidiasis
