# Supplementary material for: Genetic Epidemiology and Resistance Investigations of Clinical Yeasts in Alexandria, Egypt
Source: Pathogens. 2025 May 15;14(5):486. doi: 10.3390/pathogens14050486 (PMC12114656; doi:10.3390/pathogens14050486)
Supplement: Supplementary file 1 [file pathogens-14-00486-s001.zip › pathogens-3606172-supplementary.pdf]

**Supplementary**

**Supplementary Table S1: Isolate overview of 1307 clinical yeast isolates studied**

| Species | n |
|---|---|
| *Candida albicans* | 714 |
| *Candida tropicalis* | 274 |
| *Nakaseomyces glabratus* | 165 |
| *Pichia kudriavzevii* | 75 |
| *Candida parapsilosis* | 41 |
| *Kluyveromyces marxianus* | 11 |
| *Candida orthopsilosis* | 6 |
| *Candida dubliniensis* | 4 |
| *Pichia cactophila* | 4 |
| *Clavispora lusitaniae* | 3 |
| *Kodamaea ohmeri* | 3 |
| *Candida auris* | 2 |
| *Candida metapsilosis* | 1 |
| *Diutina rugosa* | 1 |
| *Magnusiomyces capitatus* | 1 |
| *Millerozyma farinosa* | 1 |
| *Saccharomyces cerevisiae* | 1 |

**Supplementary Table S2: Minimum inhibitory concentrations (MICs) according to CLSI M27-S4 guideline of all individual 47 bloodstream isolates.** MICs are displayed in µg/mL.

| ID | Species | AMB | FLU | ITC | VOR | POS | ISA | MFG | AFG |
|---|---|---|---|---|---|---|---|---|---|
| 232 | *Candida albicans* | 0.125 | 0.125 | ≤0.016 | ≤0.016 | ≤0.016 | ≤0.016 | 0.016 | 0.016 |
| 417 | *Candida albicans* | 0.125 | 0.125 | ≤0.016 | 0.031 | ≤0.016 | ≤0.016 | 0.031 | 0.016 |
| 519 | *Candida albicans* | 0.125 | 0.125 | ≤0.016 | ≤0.016 | ≤0.016 | ≤0.016 | 0.016 | ≤0.008 |
| 3 | *Candida parapsilosis* | 0.125 | 0.5 | ≤0.016 | ≤0.016 | ≤0.016 | ≤0.016 | 1 | 0.5 |
| 21 | *Candida parapsilosis* | 0.25 | 0.5 | ≤0.016 | ≤0.016 | ≤0.016 | ≤0.016 | 1 | 1 |
| 31 | *Candida parapsilosis* | 0.125 | 0.5 | ≤0.016 | 0.031 | ≤0.016 | ≤0.016 | 0.5 | 1 |
| 33 | *Candida parapsilosis* | 0.125 | 0.5 | ≤0.016 | ≤0.016 | ≤0.016 | ≤0.016 | 0.5 | 0.5 |
| 37 | *Candida parapsilosis* | 0.25 | 0.5 | ≤0.016 | ≤0.016 | ≤0.016 | ≤0.016 | 1 | 0.5 |
| 53 | *Candida parapsilosis* | 0.25 | 0.5 | ≤0.016 | ≤0.016 | ≤0.016 | ≤0.016 | 1 | 0.5 |
| 90 | *Candida parapsilosis* | 0.5 | 0.125 | ≤0.016 | ≤0.016 | ≤0.016 | ≤0.016 | 1 | 0.5 |
| 107 | *Candida parapsilosis* | 0.5 | 4 | 0.063 | 0.063 | ≤0.016 | ≤0.016 | 1 | 0.5 |
| 238 | *Candida parapsilosis* | 0.125 | 0.25 | 0.031 | 0.031 | 0.031 | ≤0.016 | 0.5 | 1 |
| 250 | *Candida parapsilosis* | 0.125 | 0.5 | 0.031 | 0.031 | ≤0.016 | ≤0.016 | 1 | 0.5 |
| 337 | *Candida parapsilosis* | 0.125 | 0.5 | 0.031 | 0.031 | 0.031 | ≤0.016 | 0.5 | 0.5 |

| | | | | | | | | | |
|---|---|---|---|---|---|---|---|---|---|
| 433 | *Candida parapsilosis* | 0.125 | 1 | 0.031 | 0.031 | ≤0.016 | ≤0.016 | 1 | 0.5 |
| 595 | *Candida parapsilosis* | 0.125 | 0.5 | 0.031 | ≤0.016 | ≤0.016 | ≤0.016 | 1 | 0.5 |
| 786 | *Candida parapsilosis* | 0.125 | 0.5 | 0.031 | ≤0.016 | ≤0.016 | ≤0.016 | 0.5 | 0.5 |
| 1001 | *Candida parapsilosis* | 0.125 | 0.5 | 0.031 | ≤0.016 | ≤0.016 | ≤0.016 | 0.016 | 0.031 |
| 1003 | *Candida parapsilosis* | 0.125 | 0.25 | 0.063 | 0.031 | 0.063 | ≤0.016 | 0.031 | 0.016 |
| 73 | *Candida tropicalis* | 0.063 | 0.25 | 0.016 | 0.016 | 0.016 | 0.016 | 0.016 | 0.016 |
| 100 | *Candida tropicalis* | 0.125 | 1 | 0.016 | 0.063 | 0.016 | 0.016 | ≤0.008 | 0.016 |
| 103 | *Candida tropicalis* | 0.125 | 0.25 | 0.031 | 0.031 | 0.031 | ≤0.016 | 0.031 | 0.063 |
| 110 | *Candida tropicalis* | 0.063 | 0.25 | 0.016 | 0.016 | 0.016 | 0.016 | ≤0.008 | ≤0.008 |
| 130 | *Candida tropicalis* | 0.125 | 0.25 | 0.031 | ≤0.016 | 0.031 | ≤0.016 | 0.031 | 0.031 |
| 134 | *Candida tropicalis* | 0.125 | 0.25 | 0.016 | 0.016 | 0.016 | 0.016 | ≤0.008 | ≤0.008 |
| 152 | *Candida tropicalis* | 0.031 | 0.5 | 0.031 | 0.031 | 0.016 | 0.016 | 0.016 | 0.031 |
| 182 | *Candida tropicalis* | 0.125 | 0.25 | 0.031 | ≤0.016 | 0.031 | ≤0.016 | 0.031 | 0.031 |
| 199 | *Candida tropicalis* | 0.125 | 0.5 | 0.031 | ≤0.016 | 0.031 | ≤0.016 | 0.031 | 0.031 |
| 229 | *Candida tropicalis* | 0.125 | 0.25 | 0.031 | 0.031 | 0.031 | ≤0.016 | 0.031 | 0.031 |
| 260 | *Candida tropicalis* | 0.125 | 0.25 | 0.031 | ≤0.016 | 0.031 | ≤0.016 | 0.031 | 0.016 |
| 403 | *Candida tropicalis* | 0.125 | 0.5 | 0.031 | 0.031 | 0.031 | ≤0.016 | 0.031 | 0.016 |
| 520 | *Candida tropicalis* | 0.125 | 0.25 | 0.031 | ≤0.016 | ≤0.016 | ≤0.016 | 0.031 | 0.016 |
| 601 | *Candida tropicalis* | 0.25 | 0.25 | 0.031 | 0.031 | 0.031 | ≤0.016 | 0.031 | 0.016 |
| 617 | *Candida tropicalis* | 0.125 | 0.5 | 0.031 | 0.031 | 0.031 | ≤0.016 | 0.031 | 0.016 |
| 636 | *Candida tropicalis* | 0.125 | 0.25 | 0.031 | ≤0.016 | 0.031 | ≤0.016 | 0.031 | 0.016 |
| 782 | *Candida tropicalis* | 0.125 | 0.25 | 0.031 | ≤0.016 | 0.031 | ≤0.016 | 0.031 | 0.063 |
| 983 | *Candida tropicalis* | 0.125 | 0.25 | 0.031 | 0.031 | 0.031 | ≤0.016 | 0.031 | 0.016 |
| 334 | *Kodamaea ohmeri* | 0.25 | 4 | 0.063 | 0.031 | 0.031 | ≤0.016 | 0.063 | 0.125 |
| 226 | *Millerozyma farinosa* | 0.125 | 8 | ≤0.016 | ≤0.016 | ≤0.016 | ≤0.016 | 0.016 | 0.016 |
| 4 | *Nakaseomyces glabratus* | 0.25 | 16 | 0.25 | 0.25 | 0.25 | 0.125 | 0.5 | 0.5 |
| 20 | *Nakaseomyces glabratus* | 0.25 | 2 | 0.063 | 0.031 | 0.031 | ≤0.016 | 0.031 | 0.016 |
| 119 | *Nakaseomyces glabratus* | 0.25 | 2 | 0.063 | 0.063 | 0.063 | ≤0.016 | 0.031 | 0.016 |
| 151 | *Nakaseomyces glabratus* | 0.25 | 4 | 0.125 | 0.031 | 0.125 | 0.031 | 0.031 | 0.016 |
| 312 | *Nakaseomyces glabratus* | 0.25 | 4 | 0.063 | 0.031 | 0.063 | 0.031 | 0.031 | 0.016 |
| 1002 | *Nakaseomyces glabratus* | 0.25 | 2 | 0.125 | 0.063 | 0.125 | 0.031 | 0.016 | 0.031 |
| 14 | *Pichia kudriavzevii* | 0.5 | 16 | 0.125 | 0.125 | 0.063 | 0.063 | 0.031 | 0.016 |
| 638 | *Pichia kudriavzevii* | 0.25 | 16 | 0.125 | 0.063 | 0.063 | 0.063 | 0.063 | 0.063 |

AMB, amphotericin B; FLU, fluconazole; ITC, itraconazole; VOR, voriconazole; POS, posaconazole; ISA, isavuconazole; MFG, micafungin; AFG, anidulafungin.

**Supplementary Table S3: Minimum inhibitory concentrations (MICs) according to CLSI M27-S4 guideline of 37 rare yeast isolates.** MICs are displayed in µg/mL.

| ID | Species | AMB | FLU | ITC | VOR | POS | ISA | MFG | AFG |
|---|---|---|---|---|---|---|---|---|---|
| 45 | *Candida auris* | 0.25 | 16 | 0.013 | 0.125 | 0.031 | 0.031 | 0.125 | 0.063 |
| 158 | *Candida auris* | 0.25 | 32 | 0.063 | 0.5 | 0.031 | 0.031 | 0.125 | 0.063 |
| 356 | *Candida dubliniensis* | 0.063 | 0.125 | ≤0.016 | ≤0.016 | ≤0.016 | ≤0.016 | ≤0.008 | 0.016 |
| 478 | *Candida dubliniensis* | 0.063 | 0.25 | 0.063 | ≤0.016 | 0.031 | ≤0.016 | ≤0.008 | 0.016 |

| | | | | | | | | | |
|---|---|---|---|---|---|---|---|---|---|
| 577 | *Candida dubliniensis* | 0.125 | 0.125 | 0.031 | 0.031 | ≤0.016 | ≤0.016 | 0.016 | 0.016 |
| 610 | *Candida dubliniensis* | 0.125 | 0.125 | 0.031 | ≤0.016 | ≤0.016 | ≤0.016 | ≤0.008 | ≤0.008 |
| 342 | *Candida metapsilosis* | 0.125 | 1 | ≤0.016 | ≤0.016 | ≤0.016 | ≤0.016 | 0.125 | 0.125 |
| 113 | *Candida orthopsilosis* | 0.125 | 0.125 | 0.031 | ≤0.016 | ≤0.016 | ≤0.016 | 1 | 0.5 |
| 430 | *Candida orthopsilosis* | 0.125 | 0.5 | 0.063 | ≤0.016 | 0.031 | ≤0.016 | 1 | 0.5 |
| 461 | *Candida orthopsilosis* | 0.25 | 0.5 | ≤0.016 | ≤0.016 | ≤0.016 | ≤0.016 | 1 | 0.5 |
| 463 | *Candida orthopsilosis* | 0.25 | 0.5 | 0.031 | 0.031 | ≤0.016 | ≤0.016 | 1 | 0.5 |
| 568 | *Candida orthopsilosis* | 0.125 | 0.125 | ≤0.016 | ≤0.016 | ≤0.016 | ≤0.016 | 1 | 0.5 |
| 830 | *Candida orthopsilosis* | 0.25 | 0.125 | 0.031 | ≤0.016 | 0.031 | ≤0.016 | 2 | 0.5 |
| 58 | *Clavispora lusitaniae* | 0.063 | 0.25 | ≤0.016 | ≤0.016 | ≤0.016 | ≤0.016 | 0.031 | 0.031 |
| 114 | *Clavispora lusitaniae* | 0.031 | 0.125 | ≤0.016 | ≤0.016 | ≤0.016 | ≤0.016 | 0.031 | 0.031 |
| 993 | *Clavispora lusitaniae* | 0.125 | 0.5 | ≤0.016 | ≤0.016 | ≤0.016 | ≤0.016 | 0.125 | 0.063 |
| 686 | *Diutina rugosa* | 0.25 | 2 | ≤0.016 | ≤0.016 | ≤0.016 | ≤0.016 | 0.25 | 0.25 |
| 29 | *Kluyveromyces marxianus* | 0.063 | 0.125 | ≤0.016 | ≤0.016 | ≤0.016 | ≤0.016 | 0.016 | 0.016 |
| 116 | *Kluyveromyces marxianus* | 0.125 | 0.25 | ≤0.016 | 0.031 | ≤0.016 | ≤0.016 | 0.031 | 0.063 |
| 121 | *Kluyveromyces marxianus* | 0.25 | 1 | 0.125 | 0.063 | 0.031 | ≤0.016 | 0.031 | 0.016 |
| 283 | *Kluyveromyces marxianus* | 0.25 | 1 | 0.063 | 0.031 | 0.031 | ≤0.016 | 0.031 | 0.031 |
| 414 | *Kluyveromyces marxianus* | 0.25 | 0.25 | 0.063 | ≤0.016 | 0.063 | ≤0.016 | 0.063 | 0.063 |
| 416 | *Kluyveromyces marxianus* | 0.125 | 0.25 | 0.063 | ≤0.016 | 0.031 | ≤0.016 | 0.063 | 0.063 |
| 458 | *Kluyveromyces marxianus* | 0.125 | 0.125 | ≤0.016 | ≤0.016 | ≤0.016 | ≤0.016 | 0.031 | 0.031 |
| 612 | *Kluyveromyces marxianus* | 0.25 | 0.125 | 0.063 | ≤0.016 | 0.031 | ≤0.016 | 0.031 | 0.063 |
| 640 | *Kluyveromyces marxianus* | 0.5 | 0.25 | ≤0.016 | ≤0.016 | ≤0.016 | ≤0.016 | 0.031 | 0.063 |
| 934 | *Kluyveromyces marxianus* | 0.25 | 0.125 | 0.031 | ≤0.016 | ≤0.016 | ≤0.016 | 0.031 | 0.016 |
| 995 | *Kluyveromyces marxianus* | 0.25 | 0.125 | 0.031 | ≤0.016 | ≤0.016 | ≤0.016 | 0.031 | 0.031 |
| 264 | *Kodamaea ohmeri* | 0.25 | 2 | 0.063 | 0.031 | 0.031 | ≤0.016 | 0.25 | 0.125 |
| 334 | *Kodamaea ohmeri* | 0.25 | 2 | 0.063 | 0.031 | 0.031 | ≤0.016 | 0.063 | 0.125 |
| 452 | *Kodamaea ohmeri* | 0.125 | 2 | 0.063 | 0.031 | 0.031 | ≤0.016 | 1 | 0.25 |
| 178 | *Magnusiomyces capitatus* | 0.5 | 1 | 0.063 | 0.063 | 0.063 | 0.031 | 1 | 1 |
| 226 | *Millerozyma farinosa* | 0.125 | 8 | ≤0.016 | ≤0.016 | ≤0.016 | ≤0.016 | 0.016 | 0.016 |
| 297 | *Pichia cactophila* | 0.125 | 8 | 0.063 | 0.063 | 0.031 | 0.031 | ≤0.008 | ≤0.008 |
| 391 | *Pichia cactophila* | 0.063 | 4 | 0.031 | 0.031 | ≤0.016 | ≤0.016 | ≤0.008 | ≤0.008 |
| 544 | *Pichia cactophila* | 0.063 | 8 | 0.063 | 0.063 | 0.031 | ≤0.016 | ≤0.008 | ≤0.008 |
| 474 | *Saccharomyces cerevisiae* | 0.125 | 0.5 | 0.25 | 0.063 | 0.25 | ≤0.016 | 0.25 | 0.25 |

AMB, amphotericin B; FLU, fluconazole; ITC, itraconazole; VOR, voriconazole; POS, posaconazole; ISA, isavuconazole; MFG, micafungin; AFG, anidulafungin.

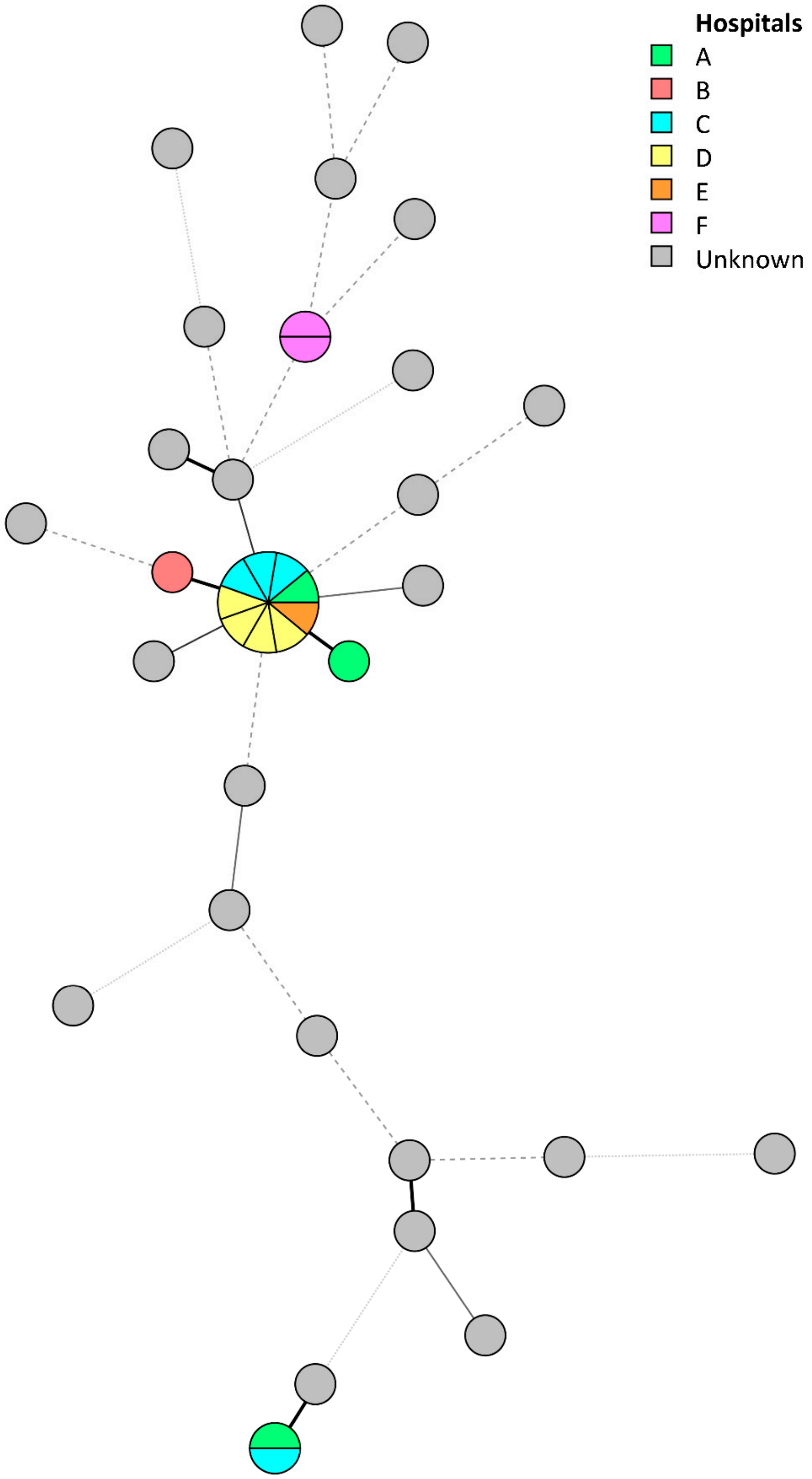


**Figure S1.** Minimum-spanning tree of 39 *Candida parapsilosis* isolates.

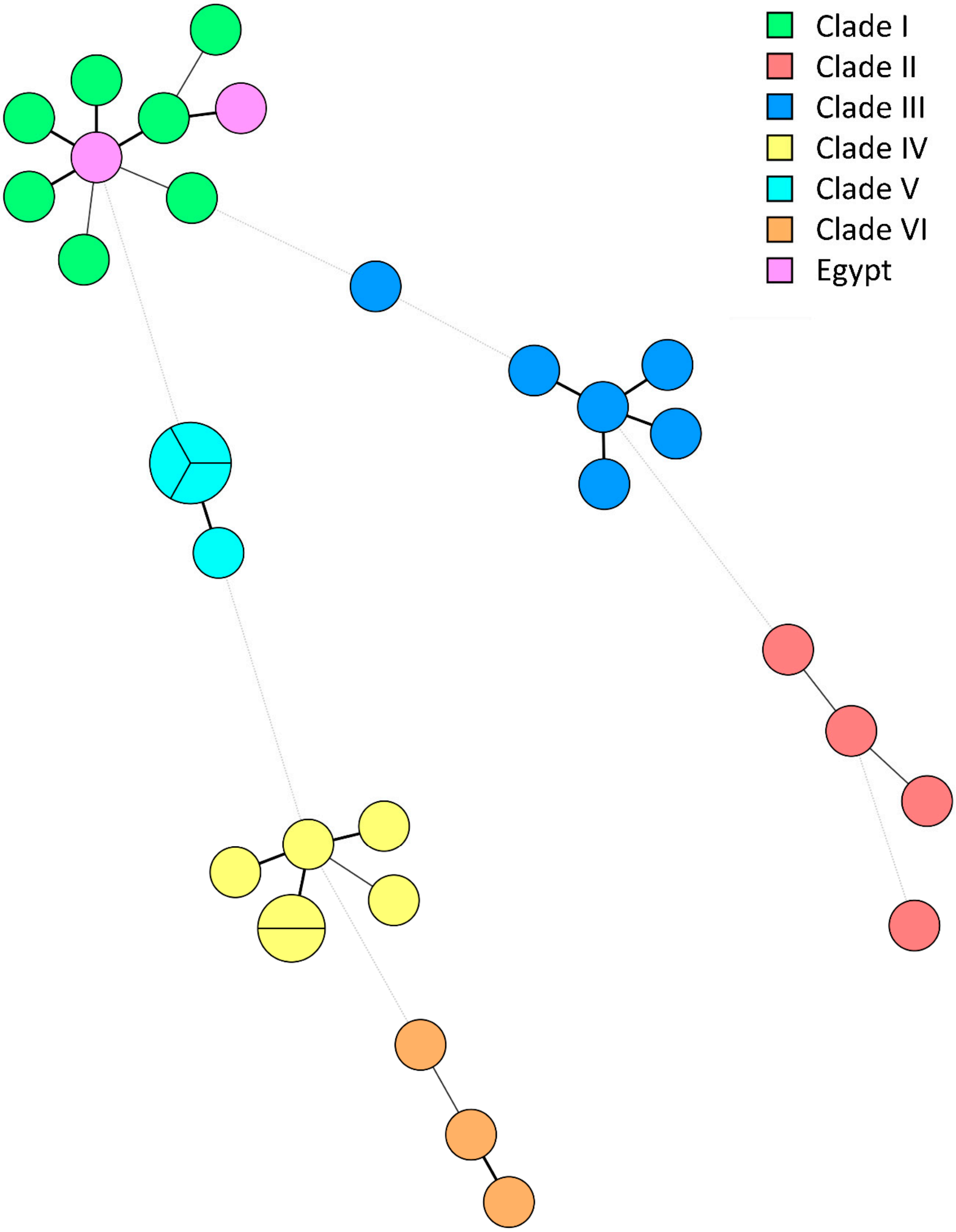


**Figure S2.** Minimum-spanning tree of two *Candida auris* isolates with control isolates from all six known clades.
